# Maternally Derived Egg Hormones, Antibodies and Antimicrobial Proteins: Common and Different Pathways of Maternal Effects in Japanese Quail

**DOI:** 10.1371/journal.pone.0112817

**Published:** 2014-11-12

**Authors:** Monika Okuliarova, Zuzana Kankova, Aline Bertin, Christine Leterrier, Erich Mostl, Michal Zeman

**Affiliations:** 1 Department of Animal Physiology and Ethology, Faculty of Natural Sciences, Comenius University, Bratislava, Slovak Republic; 2 INRA Val de Loire, UMR 85 Physiologie de la Reproduction et des Comportements CNRS-UMR 7247 - Université de Tours – IFCE, Nouzilly, France; 3 Department of Biomedical Sciences, University of Veterinary Medicine, Vienna, Austria; 4 Institute of Animal Biochemistry and Genetics, Slovak Academy of Sciences, Ivanka pri Dunaji, Slovak Republic; University of Hyderabad, India

## Abstract

Avian eggs contain a variety of maternally-derived substances that can influence the development and performance of offspring. The levels of these egg compounds vary in relation to environmental and genetic factors, but little is known about whether there are correlative links between maternal substances in the egg underlying common and different pathways of maternal effects. In the present study, we investigated genetically determined variability and mutually adjusted deposition of sex hormones (testosterone-T, androstenedione-A_4_ and progesterone-P_4_), antibodies (IgY) and antimicrobial proteins (lysozyme) in eggs of Japanese quail (*Coturnix japonica*). We used different genetic lines that were independently selected for yolk T concentrations, duration of tonic immobility and social reinstatement behaviour, since both selections for behavioural traits (fearfulness and social motivation, respectively) produced considerable correlative responses in yolk androgen levels. A higher selection potential was found for increased rather than decreased yolk T concentrations, suggesting that there is a physiological minimum in egg T levels. Line differences in yolk IgY concentrations were manifested within each selection experiment, but no consistent inter-line pattern between yolk IgY and T was revealed. On the other hand, a consistent inverse inter-line pattern was recorded between yolk IgY and P_4_ in both selections for behavioural traits. In addition, selections for contrasting fearfulness and social motivation were associated with changes in albumen lysozyme concentrations and an inverse inter-line pattern between the deposition of yolk IgY and albumen lysozyme was found in lines selected for the level of social motivation. Thus, our results demonstrate genetically-driven changes in deposition of yolk T, P_4_, antibodies and albumen lysozyme in the egg. This genetic variability can partially explain mutually adjusted maternal deposition of sex hormones and immune-competent molecules but the inconsistent pattern of inter-line differences across all selections indicates that there are other underlying mechanisms, which require further studies.

## Introduction

Avian eggs contain maternally-derived biologically active substances that have the potential to influence developmental programmes of the next generation. Through such trans-generational effects, the phenotypic variability of the progeny can be manipulated to promote rapid adaptations to prevailing environmental conditions [1]. Yolk sex steroids, mainly androgens, represent the most powerful agents that have been thoroughly studied by *in ovo* injection of exogenous hormones prior to incubation [2]. Numerous studies have shown that yolk androgen transfer into the egg is a flexible process, varying with different social and environmental conditions [3]. However, nearly the same amount of phenotypic variability of yolk testosterone (T) concentrations is explained by genetic differences among females, as was experimentally demonstrated by divergent selection for yolk T concentrations in Japanese quail (*Coturnix japonica*) [4] and also by correlative studies on similarities between mothers and daughters in small passerine birds, collared flycatchers (*Ficedula albicolis*) [5] and canaries (*Serinus canaria*) [6]. Thus, yolk androgen-mediated maternal effects can be described as indirect genetic effects with emerging evolutionary implications [7].

Experimental studies demonstrated that increased yolk androgen (T or T in combination with androstenedione-A_4_) levels influence immune functions in offspring during the early as well as later stages after hatching, but both reduced [8], [9] and enhanced [10], [11] immune responsiveness have been found. Concerning other variables, the immune-modulating effects of yolk T varied in the dose-dependent manner [12], ontogenetic development [11] and the type of immune response [13]. Beneficial effects of experimentally increased yolk T on cell mediated immunity were found using low T doses while high T dose resulted in a negative effect [12]. On the other hand, a decreased cutaneous immune response and increased bactericidal activity was demonstrated after *in ovo* T treatment in house wrens (*Troglodytes aedon*) [13]. The physiological mechanisms underlying the effects of maternal androgens on the immune system of young birds are poorly understood, but several hypotheses have been delineated. According to resource allocation theory, stimulation of energy-demanding processes (like early postnatal growth) by increased yolk androgen levels should be counterbalanced by lowered immune functions in offspring [14]. However, the results of most studies do not support such a trade-off between androgen-mediated effects on growth and immunity [8], [15]–[17], so other explanations should be considered.

In birds, the important determinants of early posthatching immune functions are provided by the maternal transfer of immune-competent substances into the eggs such as antibodies, antioxidants and antimicrobial proteins [18]. Both genetic and environmentally-driven differences in maternal transmission of these immune factors have been documented and are predicted to correlate with phenotypic variability in offspring immune responsiveness [19]–[21]. Maternally-derived yolk antibodies provide the first humoral immune protection after hatching, when the immune system is not fully developed [22]. Antioxidants in the eggs are considered to be potential inhibitors of oxidative stress, with consequences on immunity and survival of offspring [23]–[25]. Maternal antimicrobial immunity is mediated by egg lysozyme, a major antimicrobial protein, which acts through the hydrolysis of Gram-positive bacteria cell walls [26]. Thus, the correlative evolution of different pathways for maternal effects (such as maternal transfer of yolk androgens and immune factors) is expected [27]. This is supported by an inverse pattern of yolk T and IgY levels within the laying sequence in the black-headed gull, *Larus ridibundus* [27]. On the other hand, increased yolk A_4_ transfer, followed by elevated transfer of maternal antibodies, was reported in the black-legged kittiwake, *Rissa tridactyla* [28]. Taken together, there are still limited data dealing with the mutually adjusted deposition of different maternally-derived compounds in the egg, and how changes in one substance can be related to changes in others.

In the present study, we examined common and different pathways for maternal effects using genetic lines of Japanese quail that were independently selected for yolk T concentrations [4], the duration of tonic immobility (TI) and social reinstatement (SR) behaviour [29]. Lines selected for contrasting levels of yolk T, low (LET) and high (HET) egg T lines, differed also in A_4_ and estradiol in their eggs [4]. In addition, LET and HET quail displayed differences in postnatal growth [30] and some aspects of the immune response [17]. The duration of TI is a measure of fear reaction to physical restraint that exists in many species [31]. Selection for TI affected the general reactivity of birds (i.e. the propensity to be more or less frightened). Birds with the long duration of TI (LTI line) showed increased behavioural inhibition in an open field test, longer latency to enter a new environment during an emergence test and to approach novel food and were more disturbed by the sudden introduction of a frightening stimulus into their home cage as compared to quail with the short duration of TI (STI line) [32], [33]. Quail selected for high and low SR behaviour (HSR and LSR lines) differed consistently in their social motivation under various experimental conditions and in several aspects of their social behaviour such as sexual motivation or aggressive behaviour [34], [35].

Comparison of three different selection experiments provided us with an exceptional model, because lines divergently selected for the duration of TI and SR behaviour express not only corresponding differences in the level of fearfulness and social motivation, but also a correlated response in yolk androgen levels [36], [37]. In the present study, first we compared the correlative response of yolk T concentrations in lines selected for behavioural traits with direct selection for yolk T concentrations and we focused on potential physiological limitations for selection in both upward and downward directions. Second, we analysed a correlative response in deposition of immune-competent substances (yolk antibodies and albumen lysozymes) underlying mutually adjusted deposition of maternal T and these egg compounds. We hypothesized that if there was a genetically determined link between maternal transfer of T and immunological factors into the egg we would find the same pattern of differences between oppositely selected lines across all three selection experiments.

## Materials and Methods

### Animals and housing

Japanese quail from eight different genetic lines from three independent selection experiments were used in the study. Two selection programmes were conducted at the experimental unit 1295 (UE PEAT) and UMR 85, Physiologie de la Reproduction et des Comportements, INRA-CNRS-IFCE-Université de Tours, Val de Loire Centre (INRA), Nouzilly, France. [29]. The other one was conducted at the Institute of Animal Biochemistry and Genetics, the Slovak Academy of Sciences (IABG), Ivanka pri Dunaji, the Slovak Republic [4]. At the IABG, the initial population used for the selection experiment originated from a laying strain of randomly-bred Japanese quail. Birds were divergently selected for yolk T concentrations resulting in the HET and LET lines. At the INRA, the initial stock for the original breeding program was formed from two commercial strains that were reciprocally outbred. Birds were divergently selected for the duration of TI weighted for independence from SR behaviour or for SR behaviour weighted for independence from TI responses. The control lines (CTI and CSR) were derived from the same original population as the selected lines. Selection for the duration of TI was based on performance in a test, in which TI was induced by physical restraining a chick in a V-shaped cradle at 9–10 days of age. Selection for SR behaviour was based on running behaviour of 10-day-old chicks in a treadmill apparatus that reflected underlying social motivation to rejoin conspecific chicks in a goal box. For a full description of the selection and the lines, see [29].

Adult birds were housed in groups consisting of two females and one male per cage (24.5×35×20 cm) under a stimulatory light/dark cycle of either 14:10 h (IABG) or 16:8 h (INRA). Food (mash for laying hens) and water were provided *ad libitum*. During the egg collection period, one female was removed from the cage and pairs of one female and one male (3–5 months of age) remained in an individual cage to ensure female identity of eggs.

### Ethics statement

At the IABG, quails were reared in an approved breeding facility (SK PC 7010 Np) and the care and use of animals was in accordance with laws and regulations of the Slovak Republic and approved by the Ethical Committee of the Institute of Animal Biochemistry and Genetics. At the INRA, all birds were maintained at the Experimental Unit PEAT, which is registered with the Ministry of Agriculture with the license number B-37-175-1 for animal experimentation. All experiments were performed in accordance with the European Communities Council Directive 2010/63/UE and approved by ethical committees of both institutes.

### Egg collection

All eggs were collected on the day they were laid. For analysis of yolk steroid hormones, the first set of eggs was collected from 35 and 28 females from the fifth generation of the HET and LET lines, respectively (2–3 eggs per female, 182 eggs in total), then from 25, 25 and 15 females from the 53^rd^ generation of the STI, LTI and CTI lines, respectively (one egg per female, 65 eggs in total) and from 25, 25 and 15 females from the 53^rd^ generation of the HSR, LSR and CSR lines, respectively (one egg per female, 65 eggs in total). Eggs of control line in selection for contrasting yolk T concentrations were not analysed in the present study due to limited housing conditions. However, comparison of yolk T levels in the LET and HET lines with the non-selected line was reported in our previous paper [4]. Egg metrics comprising of egg, yolk and eggshell mass were recorded. The albumen mass was determined by subtracting the sum of eggshell and yolk mass from the total egg mass. Yolks were separated from the albumen, thoroughly mixed with a spatula and stored at −20°C until subsequent analysis.

For measurements of yolk immunoglobulins and albumen lysozyme, a second set of eggs was collected from 13 and 13 females from the fifth generation of the HET and LET lines, respectively (72 eggs in total), then from 11, 12 and 11 females from the 53^rd^ generation of the STI, LTI and CTI lines, respectively (102 eggs in total) and from 10, 11 and 11 females from the 53^rd^ generation of the HSR, LSR and CSR lines, respectively (96 eggs in total). On average, three eggs per female were collected. Egg metrics were recorded in the same way as for the first set of eggs. Since line differences in these egg metrics were uniform in both sets of eggs, only data from the second trial are presented. Hormones and immune components were measured in different sets of eggs since hormones were analysed in two labs according to the same protocols as previously used for respective selection lines (see below).

### Yolk hormone assay

Yolk T concentrations of quail lines from the IABG were measured after extraction by radioimmunoassay (RIA) according to the procedure described in detail by Okuliarova *et al.* [9]. A sub-sample of thawed yolks (40–45 mg) was diluted in 500 µL of deionised water and vortexed with addition of two glass beads for 3 min. Thereafter, 1500–2000 dpm of [^3^H]-testosterone were added to each sample for the individual recovery calculation (mean ± SE: 61.5±0.5%). Samples were equilibrated overnight at 4°C. They were then applied to solid phase columns filled with Extrelut NT (Merck, Darmstadt, Germany) and extracted three times, twice with 2 mL and once with 1 mL of a mixture of diethyl ether and petroleum ether (7∶3). Following evaporation under a stream of nitrogen, dried extracts were reconstituted in 300 µL of phosphate buffer (pH 7.5). Testosterone RIA was performed with 20 µL of yolk extract using [1,2,6,7-^3^H]-testosterone (PerkinElmer, USA, specific activity 63.47 Ci/mmoL) and a specific antibody generated in rabbits against testosterone-3-(carboxy-methyl) oxime bovine serum albumin conjugate. For full description of antibody cross reactivity, see [4], [38]. All samples were run in two assays with intra-assay variation coefficients of 5.5% and 7.2%, respectively. The inter-assay variation coefficient was 7.1%.

Yolk steroid (T, A_4_ and progesterone-P_4_) concentrations in quail lines from the INRA were measured after extraction by enzyme-immunoassay (EIA) according to a previously published protocol [36], [39]. The entire yolk was suspended in 10 mL of water and vortexed twice for 30 s. Samples were stored overnight at 4°C. Then, 1 mL of the mixed suspension was diluted with 4 mL of methanol, vortexed for 30 min and stored overnight at −20°C to precipitate lipids. Samples were centrifuged (−10°C, 2500 g, 10 min), and 10 µL of the supernatant was dried under a stream of nitrogen at 60°C and dissolved in 500 µL of EIA buffer. Testosterone and A_4_ were measured in 10 µL of extract diluted six times and P_4_ in 10 µL of extract diluted 66 times. For full description of antibodies and validation, see [39], [40]. Yolk T was measured in five assays, and A_4_ and P_4_ in six assays. The inter-assay variation coefficients were 19.5%, 10.4% and 15.9% for the low level pool and 12.3%, 5.5%, and 15.3% for the high level pool. The intra-assay variation coefficients were 8.5%, 4.2% and 9.2%, respectively. The hormone concentrations in the yolk are expressed in ng/g and total hormone content in the yolk is expressed in g/yolk.

### Immunoglobulin assay

Immunoglobulins were extracted from the egg yolk according to the chloroform-based protocol [41]. Briefly, 200±4 mg of thawed yolk was thoroughly vortexed with 400 µL of phosphate buffered saline (PBS) with two glass beads for 3 min. Thereafter, 600 µL of chloroform was added to the yolk homogenates, vortexed for 30 s and centrifuged at 3000×g for 15 min at 4°C. After centrifugation, an upper water layer containing proteins was transferred to a new tube and used for the analysis of IgY concentrations by enzyme-linked immunosorbent assay on 96-well plates, as described in Kankova *et al.* [17]. Plates were coated with anti-chicken IgY (Sigma-Aldrich, Cat. No. C6409) diluted 1∶800 in carbonate buffer (pH 9.6) and incubated overnight at 4°C. After washing (3×100 µl PBS/0.05% Tween 20), plates were blocked with 2.5% milk powder in PBS and incubated for 2 h at 37°C. Yolk extracts were diluted 1∶25 000 in 0.2% milk powder in PBS. Following a wash, 100 µL of diluted extracts was added to the plate and incubated for 1 h at 37°C. Chicken IgY standard (Promega, Madison, WI, USA, Cat. No. G1161) was serially diluted from 200 to 3.1 ng/mL and included on each plate. After incubation, plates were washed and incubated again with 100 µL of alkaline phosphatase conjugated anti-chicken IgY (Sigma-Aldrich, Cat. No. A9171) diluted 1∶1000 in 0.2% milk powder in Tris-buffered saline (TBS) for 1 h at 37°C. Plates were washed (3×100 µl TBS - 0.05% Tween 20) and 200 µL of p-nitrophenyl phosphate (1 mg/mL, Sigma Aldrich) in substrate buffer was added to each well and incubated at room temperature in the dark for 20 min. The reaction was stopped with 1 M sodium hydroxide. Absorbance was measured at 405 nm using a microplate reader (EL800 Bio-Tek Instruments, Winooski, VT, USA). All samples (n = 270) were measured in duplicate. Four plates were used for yolk samples from the selection for the duration of TI as well as for the selection for SR behaviour and two plates contained samples from the selection for yolk T concentrations. Mean (±SE) intra-assay variation coefficients were 3.5±1.2%, 8.0±2.3%, and 5.4±1.1%, respectively. Inter-assay variation coefficients were 5.9%, 19.7% and 6.1%, respectively. Total yolk IgY content was calculated by multiplying yolk IgY concentration (mg/g) by yolk mass (g).

### Lysozyme assay

Albumen lysozyme concentrations were analysed by the lyso-plate method [42] modified for 96-well plates [18]. Samples of albumen were diluted with deionised water (1∶7), and 10 µL of this solution was added in duplicate on the plate. Then, the wells were filled with 150 µL of a 1% agar suspension of *Micrococcus lysodeikticus* (0.5 mg/mL, Sigma-Aldrich, Cat. No. M3770). Following 60 min of incubation at room temperature, absorbance was measured at 630 nm using a microplate reader. A standard curve was prepared by serial dilutions of crystalline hen albumen lysozyme standard (Sigma-Aldrich, Cat. No. L-6876) in the range of 78–5000 µg/mL and was run on each plate to calculate lysozyme concentrations. All samples (n = 270) were measured in duplicate within seven plates. Mean (±SE) intra-assay and inter-assay variation coefficients were 7.8±1.9% and 9.3%, respectively. Total albumen lysozyme content was calculated by multiplying lysozyme concentration (mg/mL) by albumen mass (g), while conversion factor from g to mL was rounded to one.

### Data analysis

Line comparison for egg metrics, yolk sex hormone and IgY levels and albumen lysozyme concentrations was performed separately within each selection experiment. All data were examined for fit to a normal distribution by the Kolmogorov-Smirnov test. The concentration and content of yolk sex hormones in all three selection experiments, the concentration and content of albumen lysozyme in the selection for TI duration and the concentration of yolk IgY in the selection for yolk T showed a deviation from normality and thus these data were logarithmically transformed. One-way analysis of variance (ANOVA) was used to compare the concentrations and total contents of yolk steroids in lines selected for TI duration and SR behaviour. Hierarchical ANOVA (with a fixed factor of line and a random factor of female nested within line) was used to compare external egg quality parameters, yolk IgY and albumen lysozyme in all three selection experiments and yolk T levels in the lines selected for yolk T concentration. *Post hoc* comparisons were performed by Fisher's Least Significant Difference tests. The range of individual variability in yolk T levels within each selected line was demonstrated by frequency distributions of yolk T concentrations while eggs from the same female were averaged.

## Results

### Response of egg quality traits to different genetic selections

Means (± SE) of egg mass and proportions of egg metrics to total egg mass for each genetic line with corresponding statistics are shown in Table 1. The effect of female was significant in all cases, showing high inter-female variation in these egg metrics. Lines divergently selected for yolk T concentrations did not differ in any of the egg parameters.

**Table 1:** Means (± SE) of egg metrics for each genetic line of Japanese quail and corresponding among-line comparison by hierarchical ANOVA with fixed effect of line and random effect of female nested within the line.

		Egg mass (g)	Yolk/egg mass ratio (%)	Shell/egg mass ratio (%)	Albumen/egg mass ratio (%)
**Genetic line**	**HET**	9.73 (0.11)	32.0 (0.3)	8.5 (0.2)	59.5 (0.3)
	**LET**	9.34 (0.11)	31.4 (0.3)	8.6 (0.1)	60.0 (0.3)
**Line effect**	***F*** **_(1,46)_**	2.68	1.51	0.26	0.86
	***p***	0.115	0.231	0.614	0.364
**Female effect**	***F*** **_(24,46)_**	14.91	5.84	11.63	6.20
	***p***	<0.001	<0.001	<0.001	<0.001
**Genetic line**	**STI**	10.49 (0.13)^a^	30.3 (0.3)	8.4 (0.1)^a^	61.4 (0.2)
	**LTI**	11.97 (0.19)^b^	31.7 (0.2)	7.9 (0.1)^b^	60.4 (0.2)
	**CTI**	11.77 (0.14)^c^	30.7 (0.3)	8.2 (0.1)^c^	61.1 (0.3)
**Line effect**	***F*** **_(2,68)_**	8.52	2.66	3.93	1.44
	***p***	<0.01	0.086	<0.05	0.253
**Female effect**	***F*** **_(31,68)_**	30.82	9.54	7.81	7.70
	***p***	<0.001	<0.001	<0.001	<0.001
**Genetic line**	**HSR**	11.10 (0.14)^a^	32.8 (0.5)^a^	7.8 (0.1)^a^	59.4 (0.5)^a^
	**LSR**	12.03 (0.16)^b^	28.7 (0.5)^b^	7.8 (0.1)^a^	63.5 (0.5)^b^
	**CSR**	12.44 (0.11)^c^	29.1 (0.3)^b^	8.3 (0.1)^b^	62.6 (0.3)^c^
**Line effect**	***F*** **_(2,64)_**	8.20	10.15	5.19	9.35
	***p***	<0.01	<0.001	<0.05	<0.01
**Female effect**	***F*** **_(29,64)_**	21.73	7.77	11.38	7.31
	***p***	<0.001	<0.001	<0.001	<0.001

a,b,c different letters in the column indicate significant differences among lines at the level of *p*<0.05.

STI, LTI and CTI – lines selected for short and long duration of tonic immobility and their control line.

HSR, LSR and CSR – lines selected for high and low social reinstatement behaviour and their control line.

HET, LET – lines selected for high and low egg testosterone concentrations.

In lines selected for contrasting fearfulness, eggs of STI females were significantly lighter than eggs of both LTI (*p*<0.001) and CTI females (*p*<0.001), while eggs from the LTI line were heavier than those from the CTI line (*p*<0.01). On the other hand, STI and LTI eggs had the highest and the lowest proportion of shell mass, respectively (STI *vs.* LTI *p*<0.001; STI *vs.* CTI *p*<0.05; LTI *vs.* CTI *p*<0.001).

In lines selected for contrasting social motivation, eggs from the HSR line weighed less than eggs from the LSR line (*p*<0.001) and the egg mass of both selected lines was lower as compared to the non-selected CSR line (*p*<0.001 for both). HSR eggs had the highest proportion of yolk mass and the lowest proportion of albumen mass as compared to the LSR (*p*<0.001) and control lines (*p*<0.001). Eggs of lines selected for contrasting social motivation did not differ in the proportion of shell mass but both lines were lower for this parameter than non-selected controls (*p*<0.001).

### Response of yolk hormones to different genetic selections

**Figure 1:**
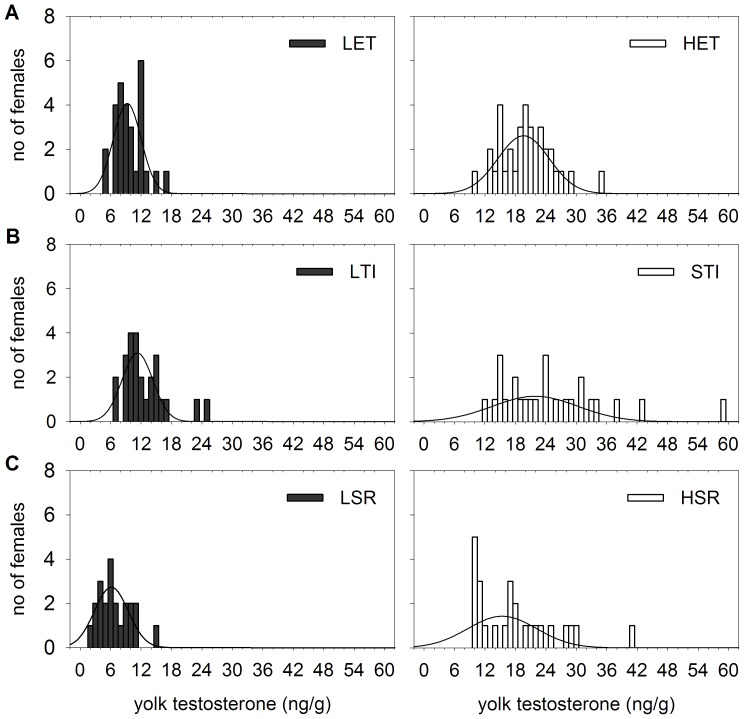
Frequency distributions of yolk testosterone (T) concentrations in genetic lines of Japanese quail. Frequency distributions of yolk T concentrations in lines selected for A) high and low egg T concentrations (the HET *vs* LET lines), B) short and long duration of tonic immobility (the STI *vs* LTI lines) and C) high and low social reinstatement behaviour (the HSR *vs* LSR lines). Fitted curves represent Gaussian regressions.

**Figure 2:**
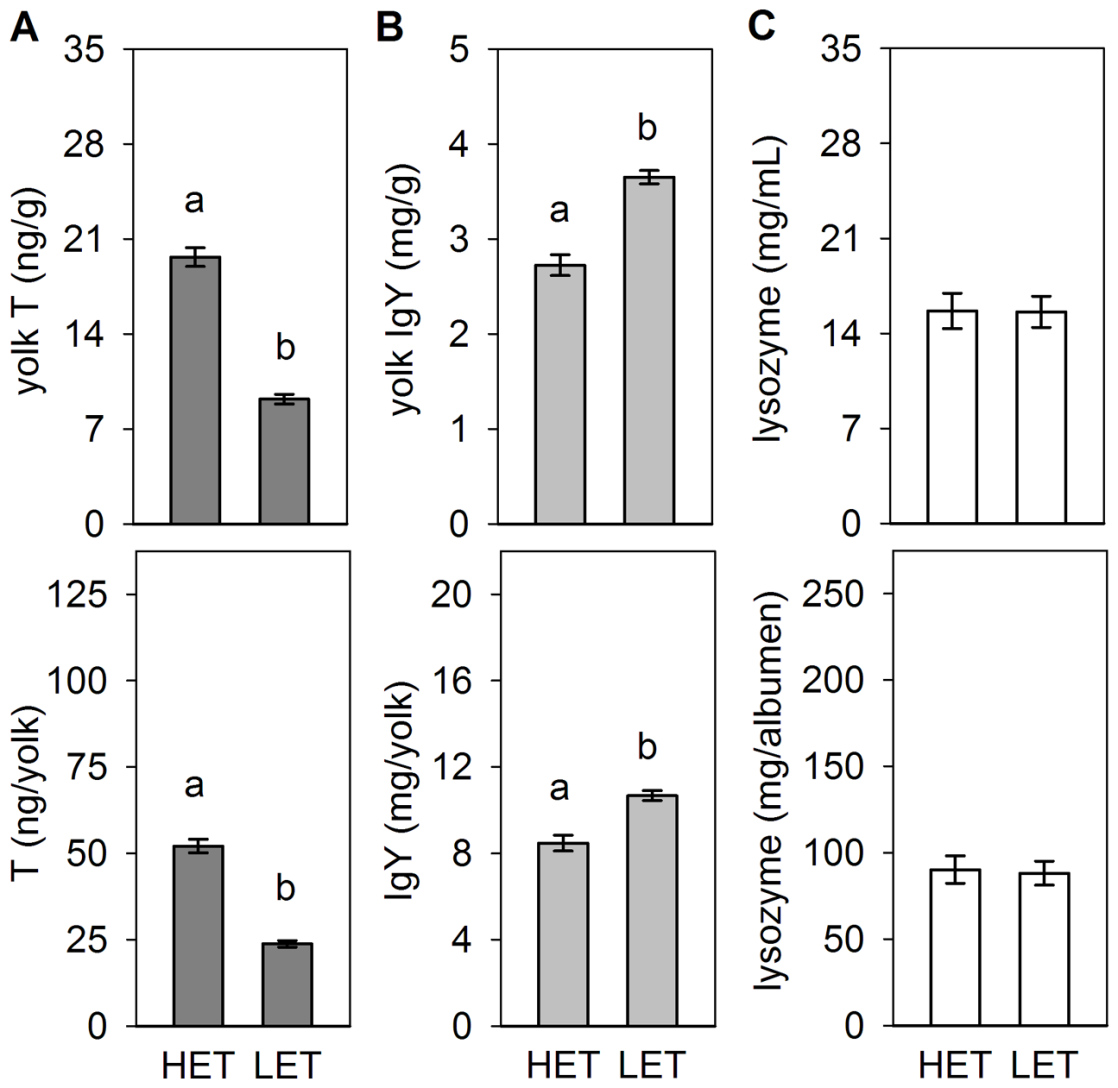
Egg compounds in Japanese quail selected for contrasting yolk testosterone (T) levels. Data represent means (± SE) of yolk T (A), yolk immunoglobulins, IgY (B) and albumen lysozyme (C) concentrations (upper row) and total egg content (bottom row) in the high (HET) and low (LET) egg T lines. Different letters above columns indicate significant differences between lines at the level of *p*<0.05.

The frequency distributions of yolk T concentrations for each genetic line are presented in Figure 1. In the LET line, female means of yolk T concentrations ranged from 4.7 to 16.2 ng/g yolk and were overlapping with those measured in the LTI (6.1–24.6 ng/g) and LSR (1.5–14.3 ng/g) lines. In the HET line, female means of yolk T concentrations ranged from 9.7 to 34.1 ng/g yolk and the upper limit was even higher in the STI (11.6–58.9 ng/g yolk) and the HSR (9.3–40.7 ng/g yolk) lines. The LET and HET lines significantly differed in yolk T concentrations (*F*_(1,116)_ = 112.24; *p*<0.001) as well as in whole yolk T content (*F*_(1,116)_ = 104.68; *p*<0.001; Fig. 2A).

**Figure 3:**
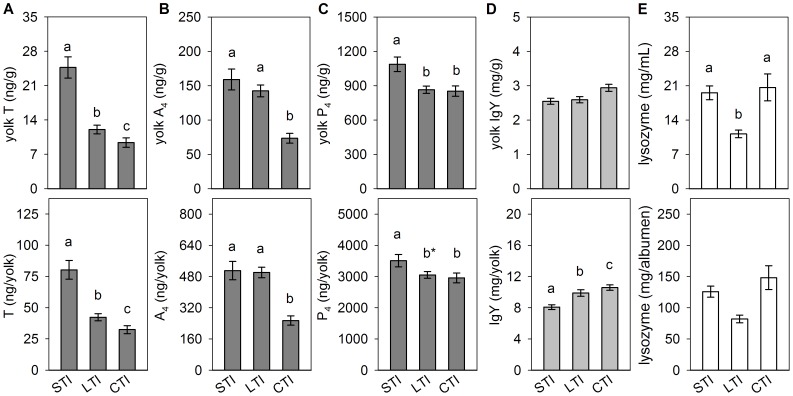
Egg compounds in Japanese quail selected for contrasting duration of tonic immobility (TI). Data represent means (± SE) of yolk T (A), yolk androstenedione, A_4_ (B), yolk progesterone, P_4_ (C), yolk immunoglobulins, IgY (D) and albumen lysozyme (E) concentrations (upper row) and total egg content (bottom row) in the lines selected for short (STI) and long (LTI) duration of TI and the non-selected control line (CTI). Different letters above columns indicate significant differences between lines at the level of *p*<0.05. b^*^ indicates *p* = 0.057.

**Figure 4:**
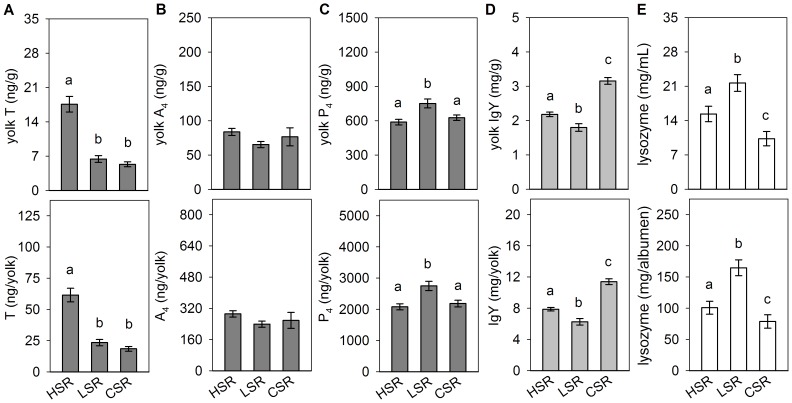
Egg compounds in Japanese quail selected for contrasting social reinstatement (SR) behaviour. Data represent means (± SE) of yolk T (A), yolk androstenedione, A_4_ (B), yolk progesterone, P_4_ (C), yolk immunoglobulins, IgY (D) and albumen lysozyme (E) concentrations (upper row) and total egg content (bottom row) in the lines selected for high (HSR) and low (LSR) SR behaviour and the non-selected control line (CSR). Different letters above columns indicate significant differences between lines at the level of p<0.05.

Mean yolk T concentrations were significantly changed by divergent selections for duration of TI (*F*_(2,62)_ = 36.86; *p*<0.001; Fig. 3A) and SR behaviour (*F*_(2,62)_ = 31.39; *p*<0.001; Fig. 4A). Eggs from STI females contained higher yolk T concentrations than eggs from LTI females (*p*<0.001) and both selected lines laid eggs with higher yolk T concentrations as compared to non-selected controls (STI *vs.* CTI *p*<0.001; LTI *vs.* CTI *p*<0.05). Females from the HSR line laid eggs with higher yolk T concentrations as compared to eggs from both the LSR (*p*<0.001) and the non-selected CSR lines (*p*<0.001), while LSR eggs did not differ from controls. The same pattern as for yolk T concentrations was found for the whole T content in the yolk (STI *vs* LTI *vs* CTI: *F*_(2,62)_ = 31.47; *p*<0.001; HSR *vs* LSR *vs* CSR: *F*_(2,62)_ = 22.36; *p*<0.001; Fig. 3A, 4A).

In lines selected for contrasting fearfulness, yolk A_4_ concentrations as well as total yolk A_4_ content did not differ between the STI and LTI eggs. However, selected lines contained higher A_4_ levels as compared to non-selected controls (for concentration: *F*_(2,62)_ = 19.58; *p*<0.001; for content: *F*_(2,62)_ = 12.13; *p*<0.001; Fig. 3B). Comparison of lines selected for contrasting level of social motivation revealed no differences in yolk A_4_ levels that as well did not differ as compared to non-selected controls (for concentration: *F*_(2,62)_ = 2.77; *p* = 0.070; for content: *F*_(2,62)_ = 1.65; *p* = 0.201; Fig. 4B).

Yolk P_4_ concentrations differed between lines selected for contrasting fearfulness (*F*_(2,62)_ = 6.76; *p*<0.01; Fig. 3C). Eggs from STI females contained higher yolk P_4_ levels than both LTI (*p*<0.01) and control CTI eggs (*p*<0.01), while LTI did not differ from controls. A similar pattern close to significance was found for yolk P_4_ content (*F*_(2,62)_ = 2.99; *p* = 0.058). In contrast to the lines selected for duration of TI, differences in yolk P_4_ levels did not reflect yolk T differences in lines divergently selected for SR behaviour (for concentration: *F*_(2,62)_ = 7.96; *p*<0.001; for content: *F*_(2,62)_ = 8.99; *p*<0.001; Fig. 4C). Eggs from LSR females contained higher yolk P_4_ levels than eggs from both HSR (*p*<0.001) and control CSR (*p*<0.05) females, while yolk P_4_ levels did not differ between the HSR and CSR lines.

### Response of yolk antibodies and albumen lysozyme to different genetic selections

Results of the statistical analysis of maternal antibodies in the yolk and albumen lysozyme are given in Table 2. The effect of female was significant in all cases, showing high inter-female variation in IgY and lysozyme deposition into the eggs.

**Table 2:** Statistical analysis (hierarchical ANOVA with fixed effect of line and random effect of female nested within the line) of maternal antibodies (IgY) in the yolk and albumen lysozyme performed separately within genetic selection for the duration of tonic immobility, social reinstatement behaviour and yolk testosterone concentrations in Japanese quail.

Selected traits	Statistics	IgY (mg/g yolk)	IgY (mg/yolk)	Lysozyme (mg/mL albumen)	Lysozyme (mg/albumen)
**Yolk testosterone**	**Line effect**	*F*_(1,46)_ = 17.56	*F*_(1,46)_ = 9.22	*F*_(1,43)_ = 0.77	*F*_(1,43)_ = 0.03
		*p*<0.001	*p*<0.01	*p* = 0.794	*p* = 0.861
	**Female effect**	*F*_(24,46)_ = 22.66	*F*_(24,46)_ = 12.43	*F*_(24,43)_ = 4.33	*F*_(24,43)_ = 4.85
		*p*<0.001	*p*<0.001	*p*<0.001	*p*<0.001
**Tonic immobility**	**Line effect**	*F*_(2,68)_ = 2.03	*F*_(2,68)_ = 4.55	*F*_(2,61)_ = 3.15	*F*_(2,61)_ = 2.15
		*p* = 0.148	*p*<0.05	*p* = 0.057	*p* = 0.133
	**Female effect**	*F*_(31,68)_ = 9.15	*F*_(31,68)_ = 10.11	*F*_(31,61)_ = 11.51	*F*_(31,61)_ = 12.75
		*p*<0.001	*p*<0.001	*p*<0.001	*p*<0.001
**Social reinstatement behaviour**	**Line effect**	*F*_(2,64)_ = 21.41	*F*_(2,64)_ = 23.86	*F*_(2,57)_ = 5.46	*F*_(2,57)_ = 5.93
		*p*<0.001	*p*<0.001	*p*<0.05	*p*<0.01
	**Female effect**	*F*_(29,64)_ = 10.01	*F*_(29,64)_ = 6.82	*F*_(29,57)_ = 8.3	*F*_(29,57)_ = 8.54
		*p*<0.001	*p*<0.001	*p*<0.001	*p*<0.001

Analysing divergent selection for yolk T concentrations, we found that HET eggs contained lower IgY levels than LET eggs (*p*<0.001 for both IgY concentrations and content; Fig. 2B). Genetic lines selected for duration of TI did not differ in yolk IgY concentrations, but significant differences were found in total IgY content (Fig. 3D). This was lower in eggs from STI than LTI females (*p*<0.001) and both lines produced eggs containing lower maternal IgY as compared to non-selected CTI eggs (STI *vs.* CTI *p*<0.001 and LTI *vs.* CTI *p*<0.01). Females from the HSR line laid eggs with higher IgY levels than eggs from the LSR line (*p*<0.001 for both IgY concentrations and content), and eggs from both selected lines contained lower IgY concentrations and total IgY content than non-selected controls (*p*<0.001 for all comparisons; Fig. 4D, Table 2).

No line differences in either albumen lysozyme concentration or content were found in quail divergently selected for yolk T concentrations (Fig. 2C). Differences in albumen lysozyme concentrations between lines selected for contrasting fearfulness approached significance, and no effect was found for the total content of albumen lysozyme (Fig. 3E). *Post hoc* comparisons revealed lower albumen lysozyme concentrations in eggs laid by LTI compared to STI (*p*<0.001) and control CTI females (*p*<0.001), while STI and CTI eggs did not differ in albumen lysozyme (Table 2). In lines selected for contrasting social motivation, both albumen lysozyme concentration and content were lower in eggs laid by HSR than LSR females (*p*<0.001; Fig. 4E). Eggs of both selected lines contained higher albumen lysozyme levels in comparison with non-selected controls (HSR *vs.* CSR *p*<0.001 for both concentrations and total content; LSR *vs.* CSR *p*<0.01 for concentrations and *p*<0.05 for total content).

## Discussion

In the present study, we focused on genetic variability and a mutual adjustment of maternally-derived sex hormones, antibodies and antimicrobial proteins in the egg. Specifically, we compared different genetic lines of Japanese quail that were obtained from bi-directional selection for yolk T concentrations and two bi-directional selections for behavioural traits (fearfulness and social motivation).

In line with previously published data [36], [37], we found that divergent selections for the duration of TI as well as for the SR behaviour resulted in a correlative response in yolk T concentrations. Therefore, we can conclude that this correlative response in T concentrations is stable and persists across generations. Indeed, since divergently selected quail lines for yolk T concentrations experimentally demonstrated genetic variation in yolk T transfer [4], all three selection experiments showed a consistent genetic component underlying inter-female differences in yolk T deposition.

Comparative analysis of three different selection strategies revealed several interesting associations that can provide a better understanding of T transfer into the egg. The pattern of the correlative response in yolk T in females selected for contrasting fearfulness and social motivation showed similar features as the direct response to selection for egg T concentrations. We recorded a limited potential for a decrease of yolk T levels as compared to the population mean in non-selected lines of Japanese quail in the present study (7.34±0.66 ng/g yolk) as well as in other studies [4], [25], [43], [44]. Moreover, a narrower range of inter-female variability in yolk T deposition was found in lines exhibiting low egg hormone levels (LTI, LSR and LET) as compared to lines with high hormone levels (STI, HSR and HET). Therefore, the data suggest that yolk T concentrations are close to their physiological minimum in these lines. An evolutionary preference for low egg T levels is not clear and may reflect the costs of high T deposition as well as a potential to increase T deposition in response to stimulation from the environment [3].

On the other hand, selections for reduced fearfulness and increased social motivation demonstrated a clear response of yolk T concentrations in an upward direction. In contrast to the lines with low egg T levels, larger variance of this yolk androgen was found in lines exhibiting high egg T concentrations (STI, HSR and HET). Moreover, a wider distribution was found in the STI and HSR quail as compared to the HET quail. Considering this wide range of inter-female variability in yolk T levels, we can assume that several genes were targeted by the selection. One set of genes could encode the enzymes of the steroidogenic pathway, since a recent study showed that aromatase mRNA expression in ovarian follicles of female house sparrows (*Passer domesticus*) is negatively correlated with yolk T concentrations in both the largest F1 follicles in the ovary and in eggs laid [45].

In spite of the different duration of selection experiments, mean egg T concentrations were comparable among the STI, HSR and HET lines suggesting that there is a common factor that limits the selection for high egg T content. Such constraints have been discussed at the level of either parental or offspring generations or both [46]. In the mother, the constraints of high egg T deposition could arise from an inability to independently regulate yolk and plasma T concentrations [47] and the resulting costs of elevated plasma T on maternal physiology and behaviour [48]. Our previous study demonstrated that selection for increased yolk T levels did not lead to a parallel increase in circulating T concentrations, suggesting independent regulation of both pools [4]. However, the costs of up-regulated steroidogenesis in females can be mediated not only through high plasma T, but also through its conversion into other active metabolites, namely estradiol [49]. In offspring, the constraints of high egg T can relate to its pleiotropic effects on diverse morphological, physiological and behavioural characteristics, resulting in potential trade-offs among these offspring traits [14]. Moreover, we found lower egg mass in lines selected for decreased fearfulness and increased social motivation as compared to their oppositely selected lines and simultaneously these line differences in egg mass were inverted to line differences in yolk T levels. In precocial birds, this inverse pattern between egg mass and yolk T levels was not reported yet and underlying mechanisms need to be further explored.

In the second part of our study, we examined the hypothesis that natural selection favours mutually adjusted deposition of different egg components, i.e. yolk androgens and yolk antibodies, to adaptively modulate trans-generational maternal effects [27], [50]. We found significant differences in yolk IgY levels between oppositely-selected lines, but these inter-line differences in yolk IgY were not consistent with the pattern of inter-line differences in yolk T deposition across the three selection experiments. Lower yolk IgY levels were detected in the STI, LSR and HET lines as compared to their oppositely-selected LTI, HSR and LET lines. Until now, only a few studies have investigated mutually related variations in yolk androgens and immunoglobulins in free living birds, and they showed inconsistent results [27], [28], [50], [51]. An inverse pattern of yolk T and IgY levels was found within the laying sequence in the black-headed gull, but there was no correlation between these egg components at the level of the laying sequence or clutches [27]. In the black-legged kittiwake, food supplemented females produced eggs with lower A_4_ and IgY levels in their replacement clutches as compared to non-supplemented females, resulting in a positive correlation between yolk A_4_ and IgY concentrations [28]. Recently, a negative correlation between yolk androgens, mainly dihydrotestosterone (DHT), and immunoglobulins was reported across individual females in the great tit, *Parus major* [50]. Taken together, it seems that there is no direct link between maternal T and antibody concentrations in the yolk that can be used to evaluate mutual adjustment of these egg components. Instead, it is likely that complex variations in other sex hormones can better reflect the female's humoral immunity and maternal antibody transmission into the yolk. In the present study, we found that eggs from the STI and LSR lines contained higher P_4_ concentrations than eggs from the oppositely-selected LTI and HSR lines. Thus, we observed the consistent inverse inter-line pattern between yolk P_4_ and IgY levels in lines selected for contrasting fearfulness and social motivation that might be explained by immunosuppressive effects of P_4_ [52]. Concerning other egg resources related to immunity, mutual adjustment of T and carotenoids was reported in Japanese quail indicating that differential allocation of maternal egg substances may depend on their mutual interactions [25].

The mechanisms beyond mutual deposition of sex hormones and immunoglobulins into the egg yolk are poorly understood but may involve both genetic and environmental components. Since we found differences in yolk IgY concentrations between oppositely selected lines of Japanese quail as well as high inter-individual variability of this trait, we can reliably expect genetic variance in maternal IgY transfer. Consistently, selection experiments for contrasting humoral immune responsiveness in hens have shown that maternal antibody transmission is to some extent genetically determined [20]. However, a different inter-line pattern between yolk T and IgY levels within each selection experiment implied that genetic correlations themselves cannot explain the mutual deposition of these egg components. Thus, at least within certain limits, maternal sex steroids and antibodies can be responsive to the same environmental and social factors, either concordantly or oppositely. Indeed, deposition of both yolk androgens and antibodies has been shown to be affected by parasitic load [53], [54], breeding density [55], [56], food supply [28], [57] and male attractiveness [58], [59].

Besides yolk immunoglobulins, egg lysozyme is another important maternally-derived immune factor that provides antimicrobial protection, not only during embryonic development, but also during the early post-hatching period [26], [60]. Albumen lysozyme concentrations were higher in the STI and LSR lines as compared to their oppositely-selected LTI and HSR lines, indicating an inverse pattern of line differences between albumen lysozyme and yolk IgY in lines selected for social motivation. No line differences in albumen lysozyme levels were detected in the selection for yolk T concentrations. Covariation in the maternal deposition of antimicrobial proteins and antibodies in the egg has already been reported, but it is not understood [61], [62]. Our results can imply genetic correlations between maternal lysozyme and antibody allocation, although we demonstrated line differences in albumen lysozyme only in two out of three selection experiments. Nevertheless, we found high variability among females which has been also detected in wild bird populations [21]. Indeed, the activity of egg lysozymes is at least partly genetically determined [63], but low heritability estimates have been calculated [64]. Therefore, environmentally-driven variation in egg lysozyme levels may prevail, as was shown for the effect of immune stimulation [65], carotenoids in the diet [19] and predation risk [62]. Moreover, we found inverse depositional patterns for lysozymes and IgY in lines selected for contrasting social motivation that may reflect compensation between different arms of the immune system (innate and humoral immune defence, respectively) but this interpretation still needs to be tested experimentally.

In conclusion, our results reveal a higher selection potential for increased rather than decreased deposition of yolk T concentrations across three independent selection experiments, suggesting that selection preserves low yolk T levels around the population mean in random-bred populations of Japanese quail. We demonstrated significant line differences in yolk IgY levels, but no consistent inter-line pattern between yolk IgY and T was found across three selection experiments. On the other hand, we recorded a consistent inverse inter-line pattern between yolk IgY and P_4_ levels in both selections for behavioural traits. In addition, selections for the duration of TI and SR behaviour related to changes in albumen lysozyme concentrations, and a negative inter-line pattern between the deposition of yolk IgY and albumen lysozyme was found in lines selected for contrasting social motivation. Thus, our data support a mutually adjusted maternal deposition of sex hormones and immune-competent molecules, although there are probably more complex interrelationships between these parameters that can by shaped not only by genetic factors. A detailed analysis of different steps of sex hormone biosynthesis is needed to better understand the co-evolution of androgens and immune substances in the egg.
